# Age‐Related Variations in Clinical and Biochemical Profiles of People Living With HIV

**DOI:** 10.1155/arat/1670270

**Published:** 2026-09-26

**Authors:** Oksana Ryabinina, Joel Adu Twum, John Anyimadu, Hilary Kenneth Addison, Paul Jerrod Amoako, Samuel Badu Nyarko, Nicholas Ekow Thomford

**Affiliations:** ^1^ Department of Chemical Pathology, School of Medical Sciences, College of Allied Health Sciences, University of Cape Coast, Cape Coast, Ghana, ucc.edu.gh; ^2^ Pharmacogenomics and Genomic Medicine Group, School of Medical Sciences, College of Health and Allied Sciences, University of Cape Coast, Cape Coast, Ghana, ucc.edu.gh; ^3^ Department of Medical Biochemistry, School of Medical Sciences, College of Health and Allied Sciences, University of Cape Coast, Cape Coast, Ghana, ucc.edu.gh; ^4^ Department of Pathology, Faculty of Health Sciences, University of Cape Town, Anzio Road Observatory, Cape Town 7925, South Africa, uct.ac.za

**Keywords:** Ghana, HIV, liver function, people living with HIV, renal function, viral suppression

## Abstract

People living with HIV (PLHIV) are at increased risk of liver and kidney dysfunction due to chronic HIV infection, long‐term antiretroviral therapy (ART), and persistent immune activation. However, age‐related differences in organ function and treatment outcomes remain insufficiently characterized in African settings. This study aimed to assess age‐related differences in liver and renal function among PLHIV in Ghana to identify patterns of organ dysfunction across different age groups. This multicenter cross‐sectional study included 558 PLHIV aged 10–76 years receiving ART, of whom 96.4% were on dolutegravir‐based regimens. Renal function was assessed using serum urea, creatinine, urea‐to‐creatinine ratio, and estimated glomerular filtration rate (eGFR). Liver function was assessed using AST/ALT ratios, hepatotoxicity grading, and fibrosis indices (APRI, FIB‐4), while virologic outcomes were assessed using HIV viral load measurements. A composite renal–hepatic–virologic abnormality score was developed based on reduced kidney function (eGFR < 60 mL/min/1.73 m^2^), elevated AST/ALT ratio (> 1), and unsuppressed viral load (≥ 1000 copies/mL). Data were analyzed using STATA, with *p* < 0.05 considered statistically significant. Renal, hepatic, and virologic parameters showed significant variation across age groups. Older participants, particularly those aged ≥ 60 years, had lower mean eGFR values (60.32 ± 17.17 mL/min/1.73 m^2^; *p* < 0.001), while adolescents aged 13–19 years had the highest frequency of virological failure (22.22%; *p* < 0.001). The composite renal–hepatic–virologic abnormality score differed significantly across age groups (*p* < 0.001), with adolescents aged 13–19 years having higher odds of a greater abnormality score compared with children aged ≤ 12 years (aOR = 3.15, 95% CI: 1.23–8.08; *p* = 0.017). In conclusion, age‐related differences were observed in renal, hepatic, and virologic abnormalities among PLHIV in Ghana. These findings support the need for age‐tailored biochemical monitoring and further longitudinal studies to clarify temporal relationships between aging and organ function in PLHIV.

## 1. Introduction

The development of antiretroviral therapy (ART) has drastically changed the prognosis of human immunodeficiency virus (HIV) infection, successfully reducing viral load and restoring immune function in people living with HIV (PLHIV) worldwide [1, 2]. Consequently, HIV infection has transitioned from a rapidly fatal illness into a chronic, manageable condition, leading to a significant and rapidly growing population of older PLHIV globally and, increasingly, in sub‐Saharan Africa (SSA) [3–6]. In Ghana, the reported national adult HIV prevalence is estimated to be around 1.7% [7]. As the population of PLHIV ages in Ghana, clinical management is increasingly shifting from acute AIDS‐defining illnesses to chronic non–AIDS‐defining morbidities (NADMs) [7]. Age modifies many aspects of HIV disease and its progression. Older individuals may present with more comorbidities, such as cardiovascular disease, renal impairment, and slower CD4 recovery, and may differ in nutritional status and metabolism compared to younger adults [2, 8, 9]. Studies from West Africa have shown that younger PLHIV often demonstrate greater immune recovery in the first year of ART compared to older individuals [10, 11]. Moreover, in aging HIV populations, “typical” biomarkers may behave differently due to age‐related changes in physiology, inflammation, liver or kidney function, or cumulative ART exposure.

The prolonged state of chronic immune activation and persistent systemic inflammation associated with HIV infection is thought to accelerate biological aging and increase the burden of age‐associated comorbidities [8, 12]. Among these NADMs, disorders of the renal and hepatic systems represent critical sources of morbidity and mortality.

Liver‐related disease, including nonalcoholic fatty liver disease (NAFLD) and viral coinfections, has emerged as a leading cause of non‐AIDS death in older PLHIV [13–17]. Hepatic function is intrinsically linked to age, with age‐related declines in liver metabolism potentially increasing the risk of hepatotoxicity from antiretroviral medications and other drugs [14, 18, 19]. Biochemical markers such as alanine aminotransferase (ALT), aspartate aminotransferase (AST), alkaline phosphatase (ALP), and bilirubin are essential for monitoring liver health and detecting drug‐induced liver injury, a key concern in long‐term ART adherence [20]. Similarly, chronic kidney disease (CKD) and various forms of HIV‐associated nephropathy are significant burdens among PLHIV, with advanced age being an established risk factor for kidney dysfunction [21, 22]. In the SSA context, the pooled prevalence of kidney dysfunction among PLHIV is high, and the widespread use of potentially nephrotoxic first‐line regimens, such as those containing tenofovir disoproxil fumarate (TDF), necessitates rigorous monitoring of renal function [23, 24]. Key renal biomarkers, including serum creatinine and urea, along with estimated glomerular filtration rate (eGFR), are fundamental tools for the early detection and management of kidney impairment [25–27].

Beyond organ‐specific biomarkers, routine clinical characteristics including immunological status, nutritional indicators, and hematological parameters may also vary with age among PLHIV and provide complementary information on health status and disease progression. However, evidence describing age‐related variation across these routinely assessed clinical and biochemical parameters remains limited in Ghana.

Although several studies in Ghana and across SSA have evaluated renal and hepatic abnormalities among PLHIV, these investigations have primarily focused on ART‐related toxicity, treatment duration, viral hepatitis coinfection, or differences by sex and treatment regimen rather than the influence of age on clinical and biochemical outcomes [21, 28–34]. As the HIV population continues to age, age has emerged as an important determinant of disease progression, treatment response, and the development of non‐AIDS comorbidities. However, little is known about whether commonly used clinical and biochemical markers differ systematically across age groups among PLHIV in Ghana [32, 35]. This represents an important knowledge gap because physiological aging, chronic HIV‐associated inflammation, and cumulative ART exposure may interact to influence liver and kidney function beyond the effects of HIV infection alone.

Characterizing differences in routine clinical and biochemical markers across age groups is important for understanding how aging influences organ function and overall health among PLHIV and for identifying individuals who may benefit from more individualized monitoring strategies. As the population of older PLHIV in Ghana continues to grow, such evidence is increasingly needed to guide age‐sensitive clinical care. Therefore, this study aimed to characterize age‐related variations in the clinical and biochemical profiles of PLHIV in Ghana, with particular emphasis on markers of liver and kidney function. The findings are expected to contribute to the evidence base needed for optimizing age‐specific monitoring and long‐term management of PLHIV in Ghana and other resource‐limited settings.

## 2. Methods

### 2.1. Study Design and Setting

This study represents a cross‐sectional analysis nested within the ongoing multicenter GenoPharm‐GH longitudinal cohort study and was conducted among PLHIV receiving care at health facilities in the Central and Western Regions of Ghana. These health facilities have designated ART centers and provide comprehensive HIV care, including clinical assessment, laboratory monitoring, and nutritional counseling. Data included in this analysis were collected between February 2019 and December 2025.

### 2.2. Study Population

The study population comprised HIV‐positive individuals who were actively enrolled in HIV care and on ART at the time of data collection. To be included in the study, participants had to be diagnosed with HIV and agree to sign a consent/assent form. Participants were recruited consecutively from ART clinics. Records of pregnant women were excluded due to the physiological changes in renal and hepatic function that occur during pregnancy, which may confound the assessment of kidney and liver parameters and limit comparability with nonpregnant participants.

### 2.3. Sample Size and Sampling Technique

This study utilized data from the ongoing longitudinal cohort. The present work represents a secondary cross‐sectional analysis of cohort participants with available clinical and biochemical data. All eligible participants meeting the inclusion criteria were included in the analysis. A total of 558 participants were available and constituted the final analytical sample.

The initial sample size estimation for the parent GenoPharm‐GH cohort [36] was performed using G∗Power 3.1 software to ensure adequate recruitment for the broader study objectives. Under an a priori assumption with a two‐tailed test with a logistic regression model, an *α* of 0.05, null hypothesis (H_0_) of 0.5, and an odds ratio (OR) of 1.3, we obtained a sample size of 473, which will give a minimum power of 0.8. However, the present analysis was not designed around a single primary liver or renal outcome and was not specifically powered for age‐stratified subgroup analyses. Consequently, all available participants were included to maximize statistical precision and representativeness across age categories.

### 2.4. Data Collection

Sociodemographic and clinical data were extracted from the GenoPharm‐GH cohort database and participant medical records using standardized data collection procedures. Variables collected included the following: sociodemographic data (age, sex, and occupation), clinical variables (duration on ART, ART regimens, systolic blood pressure [SBP], diastolic blood pressure [DBP], weight, height, etc.). Mean arterial pressure (MAP) was calculated using the standard formula: MAP = DBP + 1/3 (SBP − DBP) [37].

Data collection was conducted by trained research assistants who followed standardized protocols to ensure consistency and accuracy. Regular supervision and periodic cross‐checks were performed to verify the completeness and reliability of the collected data.

### 2.5. Anthropometric Measurements

Anthropometric measurements (weight and height) and blood pressure were taken for all participants using calibrated equipment. Weight was measured with participants wearing light clothing and no shoes, using a calibrated digital scale. Height was measured with a digital‐mounted stadiometer, Omron HBF‐702T (Krell Precision Co. Ltd), with participants standing upright without shoes. Blood pressure was measured with an automated digital blood pressure monitor (GLC‐BIG‐M6, Green Life, Canada), with participants seated comfortably, back supported, feet flat on the floor, and the left arm supported at heart level. Participants rested for at least five minutes before the first reading, and two measurements were taken at 15‐min intervals; the average of the two readings was recorded.

The blood pressures were then classified into SBP and DBP according to the WHO classification. High SBP was defined as SBP ≥ 140 mmHg, whereas high DBP was defined as a DBP ≥ 90 mmHg [38].

### 2.6. Sample Collection and Biochemical Assessments

Approximately 5 mL of venous blood was collected from each participant. The blood sample was aliquoted and used for viral loads and biochemical analysis.

Biochemical analyses were performed using the Selectra Pro XL Biochemistry analyzer (ELITech Group Clinical Systems, UK), following the manufacturer’s operating procedures and laboratory standard operating procedures (SOPs). The analyzer was routinely calibrated using manufacturer‐recommended calibrators, and preventive maintenance was conducted according to established laboratory protocols. Internal quality control (IQC) materials at normal and pathological concentration levels were analyzed daily prior to sample testing to ensure analytical accuracy and precision. Test results were accepted only when quality‐control values were within predefined acceptable ranges. The laboratory also participated in external quality assessment (EQA) and quality‐control programs to monitor assay performance and ensure compliance with established laboratory quality standards throughout the study period.

Liver function was assessed via measurement of total and direct bilirubin, ALT, AST, total protein, and albumin [39]. For renal function, serum creatinine and urea were measured, and the eGFR was calculated. For adults (≥ 18 years), eGFR was determined using the CKD‐EPI 2021 creatinine equation, whereas for participants under 18 years, the bedside Schwartz equation was used [40, 41]. Biomarkers of liver and renal assessment were determined as described in the respective package inserts and strictly according to the manufacturer’s instructions.

The AST/ALT ratio (De Ritis) and the urea‐to‐creatinine ratio were computed. APRI was calculated as (AST/AST_ULN_) × 100/platelet (10^9^/L) [42]. The FIB‐4 index was calculated as $\left(\left(\text{age}\left(\text{years}\right)\times \text{AST }\left(\text{IU}/\text{L}\right)\right)/\left(\text{platelet}\left(10^{9}/\text{L}\right)\times \sqrt{\text{ALT }\left(\text{IU}/\text{L}\right)}\right)\right)$ [43].

### 2.7. Viral Load Determination

Plasma HIV‐1 viral load was quantified using an automated COBAS AmpliPrep/COBAS TaqMan HIV‐1 Qual Test (Roche Molecular System, Inc., Branchburg, NJ, USA) following the manufacturer’s instructions. All testing was performed by trained laboratory personnel following established laboratory SOPs. The instrument underwent routine calibration, maintenance, and performance verification in accordance with manufacturer recommendations. IQC materials supplied by the manufacturer were included during testing to ensure assay accuracy and reliability, and test runs were accepted only when quality‐control results met predefined acceptance criteria. The laboratory also participated in EQA programs to monitor and maintain assay performance throughout the study period.

### 2.8. Quality Assurance

All laboratory analyses were conducted in accordance with established quality management procedures. Routine calibration, IQC, preventive maintenance, and participation in EQA programs were implemented throughout the study period to ensure analytical accuracy, precision, and reliability.

### 2.9. Variables

The primary outcomes were renal, hepatic, and virological parameters. Renal function was assessed using serum creatinine, serum urea, and eGFR. Hepatic function was evaluated using ALT, AST, total bilirubin, direct bilirubin, total protein, albumin, AST/ALT ratio, APRI, and FIB‐4 scores. Virological status was assessed using plasma HIV‐1 viral load measurements.

A composite renal–hepatic–virologic abnormality score was developed to summarize the presence of selected clinical and biochemical abnormalities among PLHIV. The score included three parameters: impaired renal function (eGFR < 60 mL/min/1.73 m^2^), abnormal liver enzyme pattern (AST/ALT > 1), and unsuppressed viral load. Each abnormal parameter contributed one point, producing a total score ranging from 0 to 3. For analysis, the score was categorized into four groups: no abnormality (score = 0), single abnormality (score = 1), two abnormalities (score = 2), and three abnormalities (score = 3).

The primary exposure variable was age group, categorized into five age groups reflecting developmental and clinical stages: children (≤ 12 years), adolescents (13–19 years), young adults (20–34 years), middle‐aged adults (35–49 years), and older adults (≥ 50 years). This categorization is commonly applied in HIV epidemiological research and surveillance systems that stratify populations into age‐specific groups to capture variations in disease burden and treatment outcomes across the life course across SSA [9, 10].

Potential confounders included sex, hypertension status, ART duration, virological status, and other available clinical characteristics known to influence renal and hepatic outcomes among PLHIV. These variables were selected based on biological plausibility and evidence from previous literature. Due to data limitations, information on viral hepatitis coinfection, alcohol use, duration of HIV infection, obesity, and concomitant medication use was not consistently available and could not be incorporated into the adjusted analyses.

Continuous biochemical variables were analyzed on their original scales. For descriptive analyses and selected regression models, certain variables were additionally categorized using established clinical thresholds, including eGFR categories, viral load suppression status, APRI risk categories, FIB‐4 risk categories, and blood pressure classifications. Age was analyzed as a categorical variable to facilitate clinically meaningful comparisons across life stages.

### 2.10. Bias

Several measures were implemented to minimize potential sources of bias. Standardized protocols were used for participant recruitment, data collection, and laboratory analyses across study sites. All biochemical and virological measurements were performed using validated automated platforms operating under established quality assurance procedures, including routine calibration, IQC, and EQA programs. To reduce measurement bias, laboratory personnel followed standardized operating procedures throughout the study period.

Potential confounding was addressed through multivariable regression analyses adjusting for available covariates. However, some relevant factors, including viral hepatitis coinfection status, alcohol consumption, cumulative ART exposure, and certain comorbid conditions, were not consistently available and therefore could not be included in the adjusted models.

Selection bias was minimized by including all eligible participants with available data from the cohort.

### 2.11. Statistical Analysis

Data were entered into a Microsoft Excel sheet, cleaned, and analyzed using Stata Version 18 (STATA Corp LLC, College Station, TX, USA).

Missing data were assessed for all study variables prior to analysis. No imputation procedures were performed. Analyses were conducted using a complete‐case approach, whereby participants with missing data for a specific variable were excluded only from analyses involving that variable. Consequently, sample sizes varied across analyses according to data availability. Patterns of missingness were reviewed and were primarily attributable to incomplete clinical records and unavailable laboratory measurements.

Descriptive statistics were used to summarize participants’ characteristics. Continuous variables were assessed for normality and presented as means ± standard deviation (SD), or medians with interquartile ranges (IQRs), as appropriate. Categorical variables were summarized as frequencies and percentages. Differences across age groups were assessed using one‐way analysis of variance (ANOVA) for normally distributed continuous variables, the Kruskal–Wallis’s test for non‐normally distributed variables, and Pearson’s chi‐square test or Fisher’s exact test for categorical variables, as appropriate.

Multivariable linear regression models were used to assess associations between age groups and continuous renal, hepatic, and virological parameters. Logistic regression models were used for binary outcomes, including reduced eGFR, elevated viral load, abnormal liver enzyme profiles, elevated APRI categories, elevated FIB‐4 categories, and other clinically categorized outcomes.

For logistic regression analyses, the constructed model was adjusted for sex. Ordinal logistic regression was used to analyze the composite score as an ordinal outcome variable. Results are presented as adjusted odds ratios (aORs) with 95% confidence intervals (CIs). A *p*‐value < 0.05 was considered statistically significant. No formal sensitivity analyses were conducted.

### 2.12. Ethical Considerations

Ethical approval for the study was obtained from the CCTH ERC (CCTHERC/EC/2020/109; CCTHERC/EC/2021/005; CCTHERC/EC/2023/090) and the Ghana Health Service Ethical Review Committee (GHS‐ERC: 001/12/24). Permission to access clinic records was obtained from facility management. All data were anonymized, and patient identifiers were removed to ensure confidentiality in accordance with ethical research standards and the principles of the Declaration of Helsinki.

## 3. Results

### 3.1. Sociodemographic Characteristics of Study Participants

A total of 580 participants’ records were screened from the GenoPharm‐GH cohort. Of these, 569 met the eligibility criteria and 11 were excluded due to pregnancy, incomplete records, or unavailable laboratory measurements. The final analysis included 558 PLHIV, with ages ranging from 10 to 76 years. The study population reflected a predominantly female (69.35%), economically active adult cohort (59.17%) with a broad age range encompassing pediatric, adolescent, and older adults (Table 1).

**TABLE 1:** Sociodemographic characteristics of study participants (*N* = 558).

Variables	Frequency (*N*)	Percentage (%)
Sex (*n* = 558)		
Male	171	30.65
Female	387	69.35
Age category, years (*n* = 558)		
≤ 12	51	9.14
13–19	63	11.29
20–39	144	25.81
40–59	243	43.55
≥ 60	57	10.22
Occupation (*n* = 455)		
Teachers	16	3.52
Farming and agriculture	38	8.35
Healthcare services	4	0.90
Security services	4	0.90
Skilled workers and services	51	11.21
Social civil services	8	1.76
Students	118	25.93
Trade and business	148	32.53
Nonworkers	39	8.57
Other	29	6.37

Regarding age distribution, most participants were in the 40–59 years of age category (43.55%), followed by those aged 20–39 years (25.81%). Adolescents (13–19 years) accounted for 11.29%, children (≤ 12 years) for 9.14%, and older adults aged ≥ 60 years represented 10.22% of the cohort. This pattern indicates that most individuals accessing HIV care at the study site were middle‐aged adults.

Occupational data were available for 455 participants. Among them, trade and business‐related occupations were the most common (32.53%), followed by students (25.93%) and skilled workers and service providers (11.21%). A smaller proportion of participants were engaged in farming and agriculture (8.35%) or were unemployed (8.57%). Occupations such as teachers, civil service, healthcare, and security services collectively accounted for less than 10% of participants, while 6.8% were categorized under other forms of work.

### 3.2. Clinical Characteristics of Participants

Clinical characteristics of study participants are represented in Table 2. Among participants with available HIV viral load data (*n* = 520), 20.58% had undetectable viral load (≤ 50 copies/mL), and 71.73% achieved virological suppression (> 50–< 1000 copies/mL), while 7.69% had virological failure (≥ 1000 copies/mL).

**TABLE 2:** Clinical and biochemical characteristics of study participants (*N* = 558).

Variables	Frequency (*N*)	Percentage (%)
HIV viral load, copies/mL (*n* = 520)		
Undetectable (≤ 50 copies/mL)	107	20.58
Virological suppression (> 50– < 1000 copies/mL)	373	71.73
Virological failure (≥ 1000 copies/mL)	40	7.69
ART duration (*n* = 285)		
≤ 4 years	202	70.88
5–9 years	47	16.49
≥ 10 years	36	12.63
ART regiments (*n* = 556)		
Dolutegravir‐based	536	96.40
Other regimens	20	3.60
SBP, mmHg (*n* = 402)		
Normal (≤ 120)	231	57.46
Pre‐HTN (121–139)	94	23.38
Hypertension (≥ 140)	77	19.15
DBP, mmHg (*n* = 371)		
Normal (≤ 80)	282	76.01
Pre‐HTN (81–89)	47	12.67
Hypertension (≥ 90)	42	11.32
MAP (*n* = 371)		
Low (≤ 70)	42	11.32
Normal (71–100)	259	69.81
High (> 100)	70	18.87
Comorbidities (*n* = 558)		
Yes	73	13.08
No	485	86.92
Comorbidities type (*n* = 73)		
NCD	33	45.21
Hematological	7	9.60
Bacterial infection	16	21.92
Fungal infection	1	1.37
Viral infection	2	2.74
Parasitic infection	6	8.22
Other	8	10.96
Total protein (*n* = 558)		
Normal (≤ 8.5 g/dL)	362	64.87
Hyperproteinemia (> 8.5 g/dL)	196	35.13
Albumin (*n* = 558)		
Normal (≥ 3.5 g/dL)	526	94.27
Hypalbuminemia (< 3.5 g/dL)	32	5.73
Albumin‐to‐globulin ratio (*n* = 558)		
Low (< 1.0)	194	34.77
Normal (1.0–2.0)	358	64.16
High (> 2.0)	6	1,08
Hepatotoxicity (*n* = 555)		
Normal, ALT < 48.0 IU/L	525	94.59
G1 (1.25–2.5 × ALT_ULN_)	28	5.05
G2 (2.5 and < 5.0 × ALT_ULN_):	1	0.18
G3 (5.0 and < 10.0 × ALT_ULN_)	1	0.18
G4: ≥ 10.0 × ALT_ULN_		
AST/ALT ratio (*n* = 552)		
Mild liver injury (< 1)	45	8.15
Chronic liver injury (1–2)	293	53.08
Significant liver disfunction (> 2)	214	38.77
APRI (*n* = 409)		
Low risk (< 0.5)	319	78.00
Moderate risk (0.5–1.5)	72	17.60
High risk (> 1.5)	18	4.40
FIB‐4 index (*n* = 408)		
Low risk (< 1.3)	200	49.02
Intermediate zone (1.3–2.67)	142	34.80
High risk (> 2.67)	66	16.18
CKD stages (*n* = 516)		
I (eGRF ≥ 90 mL/min/1.73 m^2^)	120	23.26
II (eGRF 60–89 mL/min/1.73 m^2^)	248	48.06
IIIa (eGRF 45–59 mL/min/1.73 m^2^)	97	18.80
IIIb (eGRF 30–44 mL/min/1.73 m^2^)	40	7.75
IV (eGRF 15–29 mL/min/1.73 m^2^)	6	1.16
V (eGRF < 15 mL/min/1.73 m^2^)	5	0.97
eGRF (*n* = 516)		
> 60 mL/min/1.73 m^2^	367	71.12
< 60 mL/min/1.73 m^2^	149	28.88
Renal–hepatic–virologic abnormality score (*n* = 473)		
None	32	6.77
One	282	59.62
Two	148	31.29
Three	11	2.32

*Note:* Kidney function was categorized according to the KDIGO 2012 chronic kidney disease staging system based on eGFR: Stage I, normal or high kidney function (eGFR ≥ 90 mL/min/1.73 m^2^); Stage II, mild loss of function (eGFR 60–89 mL/min/1.73 m^2^); Stage IIIa, mild‐to‐moderate loss of function (eGFR 45–59 mL/min/1.73 m^2^); Stage IIIb, moderate‐to‐severe loss of function (eGFR 30–44 mL/min/1.73 m^2^); Stage IV, severe loss of function (eGFR 15–29 mL/min/1.73 m^2^); Stage V, kidney failure (eGFR < 15 mL/min/1.73 m^2^). Normal kidney function was defined by CKD Stages 1 and 2 (with eGFR ≥ 90 mL/min/1.73 m^2^ and 60–89 mL/min/1.73 m^2^, respectively). ART—antiretroviral therapy, ALT—alanine aminotransferase, AST—aspartate aminotransferase, APRI—aspartate aminotransferase to platelet ratio index, FIB‐4—fibrosis‐4 index.

Abbreviations: CKD = chronic kidney disease, DBP = diastolic blood pressure, eGFR = estimated glomerular filtration rate, HIV = human immunodeficiency virus, MAP = mean arterial pressure, SBP = systolic blood pressure.

Information on ART duration was available for 285 participants. The majority (70.88%) had been on ART for 4 years or less, while 16.49% had received treatment for five to 9 years, and 12.63% had been on ART for 10 years or more. This distribution suggests that most participants were relatively early in their ART treatment course, with a smaller subset representing long‐term therapy recipients.

Complete data were available for 556 participants receiving ART. The majority were on a dolutegravir (DTG)–based regimen, accounting for 536 individuals (96.40%). The remaining 20 participants (3.60%) were on other ART regimens.

Blood pressure parameters were recorded for a subset of participants. Based on SBP readings (*n* = 402), more than half (57.46%) had normal SBP (≤ 120 mmHg), while 23.38% were in the prehypertensive range (121–139 mmHg) and 19.15% met the criteria for hypertension (≥ 140 mmHg). For DBP (*n* = 371), 76.01% were within the normal range (≤ 80 mmHg), 12.67% were prehypertensive (81–89 mmHg), and 11.32% were hypertensive (≥ 90 mmHg). MAP measurements (*n* = 371) further supported these findings, with 69.81% of participants falling within the normal range (71–100 mmHg), while 11.32% had low MAP (≤ 70 mmHg) and 18.87% recorded elevated MAP (> 100 mmHg).

Out of the 558 participants, only 73 (13.08%) reported having or had been clinically diagnosed with one or more comorbidities, while the majority (86.92%) had no known comorbid conditions. Among those with comorbidities, noncommunicable diseases (NCDs) were the most common, affecting 33 participants (45.21%), followed by bacterial infections in 16 participants (21.92%), hematological disorders in 7 participants (9.60%), parasitic infections in 6 participants (8.22%), viral infections in 2 participants (2.74%), fungal infection in 1 participant (1.37%), and other conditions in 8 participants (10.96%). These findings indicate that while most PLHIV in this cohort were free of comorbidities, a notable proportion had conditions that could influence clinical management and monitoring.

### 3.3. Biochemical Characteristics of Participants

Biochemical parameters, including renal and hepatic function indicators, were analyzed as continuous variables and compared across predefined age categories (Tables 2, 3, 4).

**TABLE 3:** Clinical and biochemical parameters, stratified by age group, showing trends according to age groups.

Variable	Age group					*p*‐value
	≤ 12 years mean ± SD	13–19 years mean ± SD	20–39 years mean ± SD	40–59 years mean ± SD	≥ 60 years mean ± SD	
Weight, kg: *N* = 421	30.14 ± 12.48	47.02 ± 14.40	62.12 ± 12.76	66.33 ± 16.31	64.02 ± 11.72	**<** **0.001**
MAP: *N* = 371	81.60 ± 10.96	78.40 ± 9.63	86.12 ± 15.79	94.67 ± 17.15	94.99 ± 15.01	**<** **0.001**
Platelets, × 10^9^/L: *N* = 412	256.40 ± 115.57	242.31 ± 106.60	242.83 ± 103.30	227.84 ± 91.58	214.49 ± 56.62	0.251
Renal function						
Creatinine (μmol/L): *N* = 558	67.74 ± 33.92	108.33 ± 112.21	102.79 ± 53.38	97.90 ± 62.13	109.73 ± 52.58	**0.005**
Urea (mmol/L): *N* = 514	3.67 ± 1.92	4.40 ± 3.02	3.71 ± 1.53	3.79 ± 3.07	4.28 ± 1.74	0.263
eGRF (mL/min/1.73 m^2^): *N* = 516	72.74 ± 24.97	70.98 ± 30.66	81.66 ± 26.97	73.18 ± 21.85	60.32 ± 17.17	**<** **0.001**
Liver function						
Total bilirubin (μmol/L): *N* = 552	8.88 ± 12.28	15.51 ± 26.20	11.35 ± 20.83	7.30 ± 5.09	8.09 ± 3.43	**0.001**
Direct bilirubin (μmol/L): *N* = 548	2.49 ± 3.60	3.76 ± 3.15	3.34 ± 6.85	2.50 ± 1.56	2.66 ± 1.18	0.098
Indirect bilirubin (μmol/L): *N* = 548	6.38 ± 8.98	11.75 ± 24.19	8.09 ± 14.45	4.82 ± 4.50	5.43 ± 2.77	**<** **0.001**
AST (IU/L): *N* = 556	57.64 ± 60.31	60.92 ± 74.78	39.66 ± 24.26	39.40 ± 44.05	29.72 ± 11.43	**<** **0.001**
ALT (IU/L): *N* = 555	22.20 ± 8.55	26.33 ± 18.24	23.62 ± 20.59	22.86 ± 28.91	19.66 ± 11.33	0.617
Total protein (g/dL): *N* = 558	10.45 ± 3.06	11.57 ± 2.80	9.64 ± 2.66	7.75 ± 0.86	7.69 ± 0.74	**<** **0.001**
Albumin (g/dL): *N* = 558	4.75 ± 0.98	5.00 ± 1.23	4.40 ± 0.99	4.20 ± 0.47	4.22 ± 0.41	**<** **0.001**
Globulins (g/dL): *N* = 558	5.70 ± 2.66	6.57 ± 2.31	5.24 ± 2.42	3.55 ± 0.85	3.48 ± 0.71	**<** **0.001**

*Note:* ALT—alanine aminotransferase, AST—aspartate aminotransferase. Bold values represent p values that are significant.

Abbreviations: eGFR = estimated glomerular filtration rate, MAP = mean arterial pressure.

**TABLE 4:** Clinical, biochemical, and formula‐based parameters, stratified by age groups.

Variables	Age groups					*p*‐value
	≤ 12 years *N* (%)	13–19 years *N* (%)	20–39 years *N* (%)	40–59 years *N* (%)	≥ 60 years *N* (%)	
HIV viral load, copies/mL (*n* = 520)						
Virological suppression (< 1000)	47 (92.16)	49 (77.78)	110 (85.27)	217 (98.64)	57 (100.00)	**<** **0.001**
Virological failure (≥ 1000)	4 (7.84)	14 (22.22)	19 (14.73)	3 (1.36)	0 (0)	
SBP, mmHg (*n* = 402)						
Normal (≤ 120)	22 (70.97)	45 (83.33)	77 (67.54)	74 (46.25)	13 (30.23)	**<** **0.001**
Pre‐HTN (121–139)	7 (22.58)	7 (12.96)	21 (18.42)	49 (30.63)	10 (23.26)	
Hypertension (≥ 140)	2 (6.45)	2 (3.70)	16 (14.04)	37 (23.13)	20 (46.51)	
DBP, mmHg (*n* = 371)						
Normal (≤ 80)	25 (80.65)	51 (94.44)	83 (79.81)	91 (65.47)	32 (74.42)	**0.002**
Pre‐HTN (81–89)	5 (16.13)	3 (5.56)	11 (10.58)	21 (15.11)	7 (16.28)	
Hypertension (≥ 90)	1 (3.23)	0 (0)	10 (9.62)	27 (19.42)	4 (9.30)	
MAP (*n* = 371)						
Normal (≤ 70–100)	29 (93.55)	54 (100.00)	89 (85.58)	100 (71.94)	29 (67.44)	**<** **0.001**
High (> 100)	2 (6.45)	0 (0)	15 (14.42)	39 (28.06)	14 (32.56)	
Total Protein (*n* = 558)						
Normal (≤ 8.5 g/dL)	18 (35.29)	15 (23.81)	75 (52.08)	203 (83.54)	51 (89.47)	**<** **0.001**
Hyperproteinemia (> 8.5 g/dL)	33 (64.71)	48 (76.19)	69 (47.92)	40 (16.46)	6 (10.53)	
Albumin (*n* = 558)						
Normal (≥ 3.5 g/dL)	48 (94.12)	59 (93.65)	132 (91.67)	232 (95.47)	55 (96.49)	0.554
Hypalbuminemia (< 3.5 g/dL)	3 (5.88)	4 (6.35)	12 (8.33)	11 (4.53)	2 (3.51)	
A/G ratio (*n* = 558)						
Low (< 1.0)	28 (54.09)	43 (68.25)	67 (46.53)	49 (20.16)	7 (12.28)	**<** **0.001**
Normal (1.0–2.0)	22 (43.14)	19 (30.16)	76 (52.78)	191 (78.60)	50 (87.72)	
High (> 2.0)	1 (1.96)	1 (1.59)	1 (0.69)	3 (1.23)	0 (0)	
Hepatotoxicity (*n* = 555)						
Normal, ALT < 48.0 IU/L	51 (100)	58 (93.55)	135 (94.41)	227 (93.80)	54 (94.74)	0.929
G1: ALT 48.0–119.9 IU/L	0 (0)	4 (6.45)	8 (5.59)	13 (5.37)	3 (5.26)	
G2: ALT 120.0–139.9 IU/L	0 (0)	0 (0)	0 (0)	1 (0.41)	0 (0)	
G3: ALT 240.0–479.9 IU/L	0 (0)	0 (0)	0 (0)	1 (0.41)	0 (0)	
AST/ALT ratio (*n* = 552)						
Mild liver injury (< 1)	0	4 (6.45)	15 (10.56)	21 (8.75)	5 (8.77)	**0.009**
Chronic liver injury (1–2)	22 (43.14)	26 (41.94)	72 (50.70)	136 (56.67)	37 (64.91)	
Significant liver dysfunction (> 2)	29 (56.86)	32 (51.61)	55 (37.33)	83 (34.58)	15 (26.32)	
APRI (*n* = 409)						
Low risk (< 0.5)	21 (62.63)	22 (61.11)	85 (75.36)	157 (82.20)	34 (87.18)	**0.037**
Moderate risk (0.5–1.5)	9 (28.13)	11 (30.56)	23 (20.72)	24 (12.57)	5 (12.82)	
High risk (> 1.5)	2 (6.25)	3 (14.29)	3 (2.70)	10 (5.24)	0 (0)	
FIB‐4 index (*n* = 408)						
Low risk (< 1.3)	29 (90.63)	32 (88.89)	76 (69.09)	61 (31.94)	2 (5.13)	**<** **0.001**
Intermediate zone (1.3–2.67)	1 (3.13)	1 (2.78)	26 (23.64)	89 (46.60)	25 (64.10)	
High risk (> 2.67)	2 (6.25)	3 (8.33)	8 (7.27)	41 (21.47)	12 (30.77)	
Urea‐to‐creatinine ratio (*n* = 514)						
Q1: lowest 25%	3.45–9.76	4.51–7.82	2.55–6.56	2.47–7.44	3.01–9.84	NA
Q2: 25%–50%	9.84–13.74	8.07–10.52	6.63–8.70	7.48–9.01	8.19–9.84	
Q3: 50%–75%	13.84–17.42	10.58–13.89	8.72–11.08	9.02–11.29	9.88–11.42	
Q4: highest 25%	17.84–97.23	14.12–57.31	11.12–23.86	11.32–28.31	11.61–17.48	
eGRF (*n* = 516)						
> 60 mL/min/1.73 m^2^	21 (80.77)	28 (58.33)	114 (79.72)	176 (72.73)	28 (49.12)	**<** **0.001**
< 60 mL/min/1.73 m^2^	5 (19.23)	20 (41.67)	29 (20.28)	66 (27.27)	29 (50.88)	
Renal–hepatic–virologic abnormality score (*n* = 473)						
None	0 (0)	2 (4.26)	14 (11.02)	14 (6.48)	2 (3.51)	**<** **0.001**
One	18 (69.23)	14 (29.79)	73 (57.48)	148 (68.52)	29 (50.88)	
Two	7 (26.92)	29 (61.70)	33 (25.98)	53 (24.54)	26 (45.61)	
Three	1 (3.85)	2 (4.26)	7 (5.51)	1 (0.46)	0 (0)	

*Note:* Normal kidney function was defined by CKD Stages 1 and 2 (with eGFR ≥ 90 mL/min/1.73 m^2^ and 60–89 mL/min/1.73 m^2^, respectively). A—albumin, G—globulin, ALT—alanine aminotransferase, AST—aspartate aminotransferase, APRI—aspartate aminotransferase to platelet ratio index, FIB‐4—fibrosis‐4 index. Bold values represent p values that are significant.

Abbreviations: eGFR = estimated glomerular filtration rate, HIV = human immunodeficiency virus.

Among the study population, the majority (367 participants; 71.12%) had eGFR values above 60 mL/min/1.73 m^2^, indicating normal to mildly reduced kidney function (Table 2). Conversely, 149 individuals (28.88%) had eGFR values below 60 mL/min/1.73 m^2^, consistent with moderate‐to‐severe impairment. Based on eGFR‐derived KDIGO staging, nearly half of the participants were classified as CKD Stage II (60–89 mL/min/1.73 m^2^), totaling 248 individuals (48.06%). Stage I CKD (≥ 90 mL/min/1.73 m^2^) accounted for 120 participants (23.26%). Moderate kidney impairment was represented by Stage IIIa in 97 participants (18.80%) and Stage IIIb in 40 participants (7.75%). Severe reductions in kidney function were less common, with 6 individuals (1.16%) in Stage IV and 5 individuals (0.97%) in Stage V renal failure. Renal function markers, including creatinine, urea, and eGFR, were assessed across different age groups (Tables 3 and 4).

Age‐stratified analysis of eGFR revealed significant differences across the five age groups (*p* < 0.001). Many participants in the younger age groups had preserved kidney function (eGFR > 60 mL/min/1.73 m^2^), including 80.77% of children aged ≤ 12 years, 58.33% of adolescents (13–19 years), and 79.72% of adults aged 20–39 years. Reduced kidney function (eGFR < 60 mL/min/1.73 m^2^) was more prevalent among older participants, particularly those aged ≥ 60 years (50.88%) and 40–59 years (27.27%), compared to younger age groups (≤ 12 years: 19.23%; 20–39 years: 20.28%). These findings indicate a clear age‐related decline in renal function (Table 4).

Age was significantly associated with HIV virological outcomes (*p* < 0.001). Virological suppression was highest among patients aged ≥ 60 years (100.0%) and those aged 40–59 years (98.64%), while the lowest suppression rate was observed among adolescents aged 13–19 years (77.78%). Conversely, virological failure was most frequent among adolescents (22.22%) and young adults aged 20–39 years (14.73%), with no cases observed among patients aged ≥ 60 years (Table 4). These findings indicate substantial age‐related differences in viral load control, with poorer outcomes observed among children, adolescents, and young adults compared with older adults.

The AST/ALT ratio showed significant variation across age categories (*p* = 0.009). An AST/ALT ratio > 2, which may indicate a pattern of transaminase elevation associated with certain hepatic conditions, was most common among children aged ≤ 12 years (56.86%) and adolescents aged 13–19 years (51.61%), and progressively declined with increasing age, reaching 26.32% in participants aged ≥ 60 years. Ratios between 1 and 2 were the most frequent across all groups and increased steadily with age, from 43.14% in the youngest group to 64.91% among those aged more than 60 years. Ratios below 1 were relatively uncommon overall and occurred predominantly in participants aged 13–19 years (6.45%) and 20–39 years (10.56%).

Age‐specific comparisons showed statistically significant variation in total protein levels across the five age groups (*p* < 0.001). Hyperproteinemia was markedly more common among children (≤ 12 years; 64.71%) and adolescents (13–19 years; 76.19%) compared to adults aged 40–59 years (16.46%) and those ≥ 60 years (10.53%). Conversely, normal total protein levels were more frequent in older age groups, reaching 89.47% among participants aged ≥ 60.

Statistical analysis indicated a significant difference in APRI risk categories across age groups (*p* = 0.037), suggesting variation in liver fibrosis risk with age. FIB‐4 increased across age groups. However, interpretation should be made cautiously because age is incorporated directly into the FIB‐4 calculation. Most children (< 12 years) and adolescents (13–19 years) were classified as low risk (< 1.3), 90.63% and 88.89%, respectively, whereas only 31.94% of middle‐aged adults (40–59 years) and 5.13% of older adults (≥ 60 years) fell in the low‐risk category. Correspondingly, intermediate (1.3–2.67) and high‐risk (> 2.67) proportions increased with age, particularly among adults ≥ 40 years. The difference in FIB‐4 risk categories across age groups was highly significant (*p* < 0.001), indicating an age‐associated increase in estimated liver fibrosis in this cohort.

Logistic regression analysis examining the association between age groups and selected virological, liver, and renal biomarkers among PLHIV was undertaken (Table 5). Both crude ORs and gender‐adjusted OR were estimated. Compared with children aged ≤ 12 years, adolescents aged 13–19 years had significantly higher odds of unsuppressed viral load (aOR = 3.48; 95% CI: 1.06–11.38; *p* = 0.039). However, no significant association was observed for participants aged 20–39 years (aOR = 2.25; 95% CI: 0.71–7.11; *p* = 0.169). Participants aged 40–59 years had significantly lower odds of unsuppressed viral load compared with the reference group (aOR = 0.19; 95% CI: 0.04–0.88; *p* = 0.033). Participants aged 20–39 years showed lower odds of hyperproteinemia in the crude model (OR = 0.50; 95% CI: 0.26–0.97*;* *p* = 0.041), although this association was no longer statistically significant after adjustment for gender (aOR = 0.54; 95% CI: 0.28–1.06; *p* = 0.073). Participants aged 40–59 years and those aged ≥ 60 years had significantly lower odds of high total protein levels compared with the reference group even after adjustment (aOR = 0.12; 95% CI: 0.06–0.23; *p* < 0.001 and aOR = 0.07; 95% CI: 0.02–0.19; *p* < 0.001, respectively). No statistically significant association was observed between age groups and low albumin levels and high albumin–globulin (A/G) ratio in both crude and adjusted models (*p* > 0.05). Similarly, no significant associations were found between age groups and hepatotoxicity and AST/ALT ratio more than 1 (*p* > 0.05). Age groups were not significantly associated with the high‐risk APRI or FIB‐4 in both crude and adjusted models (*p* > 0.05). Renal impairment showed significant variation across age groups. Participants aged ≥ 60 years had significantly lower odds of reduced renal function compared with the reference group (aOR = 0.23; 95% CI: 0.08–0.69; *p* = 0.009). No statistically significant differences were observed for other age groups.

**TABLE 5:** Logistic regression of categorical clinical and biochemical markers by age groups among PLHIV.

Outcome variable	Age group	OR (95% CI)	*p*‐value	aOR (95% CI)	*p*‐value
HIV viral load (copies/mL)	≤ 12 years	1 (ref)	—	1 (ref)	—
	13–19 years	3.36 (1.03–10.94)	**0.044**	3.48 (1.06–11.38)	**0.039**
	20–39 years	2.03 (0.65–6.29)	0.220	2.25 (0.71–7.11)	0.169
	40–59 years	0.16 (0.04–0.75)	0.020	0.19 (0.04–0.88)	**0.033**
	≥ 60 years	1	—	1	—

MAP	≤ 12 years	1 (ref)	—	1 (ref)	—
	13–19 years	1	—	1	—
	20–39 years	2.44 (0.53 = 11.33)	0.254	2.39 (0.51 = 11.14)	0.267
	40–59 years	5.66 (1.29–24.84)	**0.022**	5.47 (1.23–24.31)	**0.026**
	≥ 60 years	7.00 (1.46–33.59)	**0.015**	6.86 (1.42–33.06)	**0.016**

Total protein (g/dL)	≤ 12 years	1 (ref)	—	1 (ref)	—
	13–19 years	1.75 (0.77–3.95)	0.181	1.79 (0.79–4.06)	0.164
	20–39 years	0.50 (0.26–0.97)	**0.041**	0.54 (0.28–1.06)	0.073
	40–59 years	0.11 (0.06–0.21)	**<** **0.001**	0.12 (0.06–0.23)	**<** **0.001**
	≥ 60 years	0.07 (0.02–0.19)	**<** **0.001**	0.07 (0.02–0.19)	**<** **0.001**

Albumin (g/dL)	≤ 12 years	1 (ref)	—	1 (ref)	—
	13–19 years	1.08 (0.23–5.08)	0.918	1.13 (0.24–5.33)	0.874
	20–39 years	1.45 (0.39–5.38)	0.574	1.70 (0.45–6.44)	0.437
	40–59 years	0.76 (0.20–2.82)	0.680	0.91 (0.24–3.50)	0.890
	≥ 60 years	0.58 (0.09–3.63)	0.562	0.66 (0.10–4.14)	0.553

A/G ratio	≤ 12 years	1 (ref)	—	−1 (ref)	—
	13–19 years	0.87 (0.05–14.27)	0.919	0.87 (0.05–14.27)	0.919
	20–39 years	0.45 (0.03–7.71)	0.582	0.45 (0.03–7.71)	0.582
	40–59 years	0.85 (0.08–9.15)	0.892	0.85 (0.08–9.15)	0.892
	≥ 60 years	—	—	—	—

ALT (IU/L)	≤ 12 years	1 (ref)	—	1 (ref)	—
	13–19 years	1.15 (0.25–5.80)	0.856	1.15 (0.25–5.80)	0.856
	20–39 years	1.10 (0.28–4.36)	0.887	1.10 (0.28–4.36)	0.887
	40–59 years	1.27 (0.35–4.57)	0.716	1.27 (0.35–4.57)	0.716
	≥ 60 years	—	—	—	—

AST/ALT ratio	≤ 12 years	1 (ref)	—	1 (ref)	—
	13–19 years	1.39 (0.36–5.43)	0.634	1.28 (0.33–5.08)	0.721
	20–39 years	0.81 (0.28–2.36)	0.704	0.84 (0.29–2.44)	0.752
	40–59 years	1.00 (0.36–2.78)	0.996	1.07 (0.38–2.98)	0.900
	60+ years	—	—	—	—

FIB‐4 index	≤ 12 years	1 (ref)	—	1 (ref)	—
	13–19 years	1.55 (0.19–12.67)	0.682	1.55 (0.19–12.67)	0.682
	20–39 years	0.76 (0.13–4.39)	0.759	0.76 (0.13–4.39)	0.759
	40–59 years	2.43 (0.52–11.41)	0.260	2.43 (0.52–11.41)	0.260
	≥ 60 years	4.16 (0.82–21.18)	0.086	4.16 (0.82–21.18)	0.086

APRI	≤ 12 years	1 (ref)	—	1 (ref)	—
	13–19 years	0.87 (0.12–6.19)	0.892	0.87 (0.12–6.19)	0.892
	20–39 years	0.39 (0.08–1.93)	0.245	0.39 (0.08–1.93)	0.245
	40–59 years	0.47 (0.11–1.95)	0.297	0.47 (0.11–1.95)	0.297
	≥ 60 years	—	—	—	—

eGFR (< 60 mL/min/1.73 m^2^)	≤ 12 years	1 (ref)	—	1 (ref)	—
	13–19 years	0.33 (0.11–1.03)	0.057	0.33 (0.11–1.03)	0.057
	20–39 years	0.93 (0.32–2.70)	0.897	0.93 (0.32–2.70)	0.897
	40–59 years	0.63 (0.23–1.76)	0.379	0.63 (0.23–1.76)	0.379
	≥ 60 years	0.23 (0.08–0.69)	**0.009**	0.23 (0.08–0.69)	**0.009**

Renal–hepatic–virologic abnormality score	≤ 12 years	1 (ref)	—	1 (ref)	—
	13–19 years	3.15 (1.23–8.07)	**0.017**	3.15 (1.23–8.08)	**0.017**
	20–39 years	0.77 (0.34–1.78)	0.549	0.80 (0.35–1.86)	0.609
	40–59 years	0.65 (0.29–1.45)	0.293	0.69 (0.31–1.53)	0.358
	≥ 60 years	1.46 (0.59–3.58)	0.414	1.50 (0.61–3.69)	0.382

*Note:* A—albumin, G—globulin, ALT—alanine aminotransferase, AST—aspartate aminotransferase, APRI—aspartate aminotransferase to platelet ratio index, FIB‐4—fibrosis‐4 index. aOR = odds ratio adjusted for sex. Bold values represent p values that are significant.

Abbreviations: eGFR = estimated glomerular filtration rate, HIV = human immunodeficiency virus.

### 3.4. Age‐Related Variation in Renal–Hepatic–Virologic Abnormality Score

Among the 473 participants, most had one renal–hepatic–virologic abnormality (282, 59.62%), followed by two abnormalities (148, 31.29%). Only 32 participants (6.77%) had no abnormalities, while 11 (2.32%) had abnormalities in all three domains (Table 2).

The distribution of the renal–hepatic–virologic abnormality score varied across age groups (Table 4). Participants aged 13–19 years had the highest proportion with two abnormalities (29/47, 61.70%), followed by those aged ≥ 60 years (26/57, 45.61%). In contrast, participants aged 40–59 years had the highest proportion with one abnormality (148/216, 68.52%). The distribution of the composite abnormality score differed significantly across age groups (*χ* ^2^ test, *p* < 0.001).

Ordinal logistic regression analysis showed that adolescents aged 13–19 years had significantly higher odds of having a higher renal–hepatic–virologic abnormality score than children aged ≤ 12 years (aOR = 3.15, 95% CI: 1.23–8.08; *p* = 0.017). Although participants aged ≥ 60 years had higher adjusted odds of a higher abnormality score than the reference group, the association was not statistically significant (aOR = 1.50, 95% CI: 0.61–3.69; *p* = 0.382) (Table 5).

### 3.5. Overlap of Renal, Hepatic, and Virological Abnormalities

Among the 473 participants with complete data for all three variables, the overlap of reduced kidney function (eGFR < 60 mL/min/1.73 m^2^), elevated AST/ALT ratio (> 1), and unsuppressed HIV viral load is presented in Supporting Figure S1. Thirteen participants (2.75%) had none of the three abnormalities. Elevated AST/ALT ratio (> 1) was the most common isolated abnormality, occurring in 122 participants (25.79%). Isolated unsuppressed HIV viral load was observed in 20 participants (4.23%), whereas isolated reduced kidney function (eGFR < 60 mL/min/1.73 m^2^) was uncommon, occurring in only 3 participants (0.63%).

Co‐occurrences of abnormalities were frequent. The combination of elevated AST/ALT ratio and unsuppressed HIV viral load, in the absence of reduced kidney function, was the most common pattern, occurring in 176 participants (37.21%). Reduced kidney function and elevated AST/ALT ratio without unsuppressed viral load were observed in 61 participants (12.90%), while reduced kidney function and unsuppressed viral load without elevated AST/ALT ratio were identified in 7 participants (1.48%). All three abnormalities co‐occurred in 71 participants (15.01%).

## 4. Discussion

This study identified important differences in clinical and biochemical profiles across age groups among PLHIV. Younger participants generally had better hepato‐ and renal function based on their eGFR, AST/ALT, APRI, and FIB‐4 but experienced more virologic failures. Conversely, adults (≥ 50 years) have reduced hepato‐ and renal function but can achieve significant viral suppression (< 1000 copies/mL). In Ghana, an estimated 334,000 people are living with HIV, with adult prevalence among those aged 15–49 years around 1.5% and notable sex and regional differences in distribution, including higher prevalence among women and young people [44]. This study is comparable to other works where similar age gradients in virologic outcomes have been consistently reported across SSA, where younger PLHIV remain disproportionately affected by suboptimal viral suppression despite widespread ART availability [45, 46].

The elevated prevalence of virological failure observed among children and adolescents may be attributed to a range of biological, treatment‐related, and psychosocial factors. Children living with HIV often depend on caregivers for medication, and inconsistent caregiving, delayed disclosure of HIV status, and limited pediatric‐friendly formulations can adversely affect adherence [47, 48]. Adolescents face additional challenges, including treatment fatigue, stigma, mental health concerns, and difficulties during transition from pediatric to adult HIV care services, all of which have been linked to poorer virologic outcomes [45, 49]. Studies from Ghana and other African countries have similarly reported lower rates of viral suppression among young adults compared with older PLHIV, even in the context of effective national ART programs [30, 50].

The lower odds of elevated viral load observed among older adults should be interpreted cautiously. Although age‐group differences were identified, the cross‐sectional nature of the study precludes determination of whether age itself contributed to improved virological outcomes. Alternative explanations may include greater treatment adherence, longer engagement in HIV care, cumulative ART exposure, enhanced healthcare utilization, and survivor effects among older individuals who remain in care. Because these factors were not comprehensively assessed in the present study, the mechanisms underlying the observed associations remain uncertain and require further investigation through longitudinal studies.

In contrast, older adults in this study demonstrated markedly higher levels of virological suppression and minimal virological failure. This finding is consistent with evidence from African and global cohorts, showing that older PLHIV tend to have better adherence, longer duration on ART, and more stable engagement in care, contributing to improved virologic control [49, 51]. Prolonged exposure to ART and sustained viral suppression among older adults may also partly explain the observed burden of renal impairment in this group, as kidney dysfunction may reflect cumulative injury accrued during earlier periods of uncontrolled viremia and long‐term treatment exposure rather than ongoing viral replication, especially as one of the components of the DTG‐based ART, tenofovir, has previously been shown to affect renal function [52–55]. However, the persistence of virological failure among younger age groups underscores the need for age‐specific interventions, including intensified adherence support, youth‐friendly services, and more frequent viral load monitoring, to sustain long‐term treatment success and prevent downstream complications such as CKD.

The pathogenesis of renal impairment in PLHIV is multifactorial reflecting the combined effects of aging, genetic susceptibility, HIV‐related kidney disease, comorbidities and coinfections, and cumulative exposure to ART [56, 57].

Consistent with established physiological processes, eGFR decreased across successive age groups, with the lowest values observed among older participants. Age‐related decline in renal function has been widely documented in the general population and is characterized by progressive reductions in glomerular filtration rate over time (REF). Because the present study did not include an HIV‐negative comparison group, it is not possible to determine the extent to which the observed differences reflect normal aging, HIV‐related factors, long‐term ART exposure, or a combination of these influences. Nevertheless, the findings indicate that older PLHIV may represent a population requiring closer monitoring of renal function. Age‐related reductions in eGFR are well recognized in the general population and should therefore be considered when interpreting differences observed across age groups in this study.

Similar patterns have been observed in African and European cohorts of PLHIV, where older age groups generally exhibited lower eGFR values and higher serum creatinine concentrations [58–62]. These findings support the observation that renal biomarkers differ across age groups among PLHIV, although the relative contributions of biological aging, HIV‐related factors, and cumulative ART exposure remain uncertain.

Given the widespread adoption of DTG‐based regimens in Ghana, interpretation of renal impairment in contemporary cohorts of PLHIV is complex. Renal dysfunction may reflect a combination of factors, including age‐related changes, prior ART exposure, HIV‐associated renal disease, comorbid conditions, and preexisting kidney injury acquired before initiation of current treatment regimens.

The prevalence of decreased kidney function, as indicated by reduced eGFR, was higher in our cohort than that reported in studies from Burundi, Ethiopia, and South Africa, suggesting heterogeneity in ART duration, regimen composition, comorbid disease burden, and access to routine renal monitoring across settings [63]. While long‐term exposure to TDF and protease inhibitors has historically been associated with progressive renal dysfunction, the widespread transition to DTG‐based regimens has seemingly altered this risk profile [64]. In Ghana and similar settings, where DTG is now the preferred first‐line therapy, interpretation of reduced eGFR among PLHIV should consider prior ART exposure, HIV‐associated renal disease, age‐related changes, comorbid conditions, and preexisting kidney injury. Although DTG has not been consistently associated with clinically significant declines in eGFR, the cross‐sectional design of the present study does not permit evaluation of the temporal relationship between ART exposure and renal function.

Recent evidence indicates that CKD prevalence among young PLHIV in SSA remains substantial. A 2024 systematic review reported a pooled CKD prevalence of 12.0% among young PLHIV in SSA, with wide heterogeneity ranging from 0.8% to 53% [65, 66]. African studies among children aged ≤ 18 years have reported CKD prevalence ranging from 6.7% to 34.6%. In our cohort, 19.23% of children aged ≤ 12 years and 41.67% of adolescents aged 13–19 years had eGFR values < 60 mL/min/1.73 m^2^, highlighting the burden of reduced kidney function markers among younger PLHIV.

Although the cross‐sectional design does not permit determination of the underlying causes or progression of renal dysfunction, these findings support the importance of routine renal monitoring in pediatric and adolescent HIV care, particularly given the potential for prolonged lifetime exposure to ART and other risk factors affecting kidney health.

The liver plays a central role in the biotransformation and detoxification of xenobiotics, rendering hepatocytes particularly vulnerable to drug‐induced liver injury. In PLWH, this susceptibility is amplified by chronic immune activation, direct viral effects, coinfections, and long‐term exposure to ART. Although newer antiretroviral regimens are generally more potent and better tolerated than earlier generations, hepatotoxicity remains one of the most clinically significant and potentially life‐threatening complications associated with ART. In this study, liver morbidity was assessed using conventional liver enzymes alongside derived and noninvasive fibrosis indices, including ALT, AST, AST/ALT ratio, APRI, FIB‐4, and grading of hepatotoxicity. These surrogate markers are particularly valuable in resource‐limited settings where liver biopsy and advanced imaging are not routinely available.

We observed that elevations in AST were more frequent than elevations in ALT, while serum albumin levels remained relatively stable across age groups, reflecting that mild AST elevation may result from nonhepatic factors such as muscle metabolism, immune activation, or subclinical hepatic stress rather than overt hepatocellular injury [32]. Mild transaminase abnormalities are common in PLWH, even in the absence of viral hepatitis coinfection, and do not always correlate with clinically apparent liver disease. Overall hepatotoxicity, defined by ALT elevation, was low (5.41%), predominantly Grade 1, and no Grade 4 hepatotoxicity was observed, supporting the hepatic safety of contemporary ART regimens, particularly in participants with shorter treatment duration (≤ 4 years: 70.88%). Evidence from African cohorts suggests that cumulative exposure to DTG‐based regimens may be associated with mild transaminase elevations over time, reinforcing the need for ongoing liver monitoring [32, 67]. Previous studies from SSA and other regions report that most ART‐associated liver enzyme abnormalities are transient and clinically insignificant in the absence of viral hepatitis coinfection or advanced liver disease [32, 67]. Data from Ethiopia and Uganda similarly demonstrate low rates of severe hepatotoxicity among patients receiving modern ART regimens, including DTG‐based therapy [68, 69].

The AST/ALT ratio is commonly used as a nonspecific indicator of patterns of liver enzyme abnormalities rather than a definitive measure of liver dysfunction or fibrosis. In the present study, nearly half of the participants exhibited AST/ALT ratios greater than 2, indicating a predominance of AST relative to ALT. However, this finding should be interpreted cautiously because elevated AST/ALT ratios may arise from a variety of hepatic and extrahepatic factors, including age‐related physiological changes, muscle metabolism, chronic inflammation, medication exposure, and underlying liver disease. In the absence of imaging‐based assessments or histological confirmation, the clinical significance of these elevated ratios cannot be determined. Pediatric studies suggest that adult cutoffs may underestimate pathology in children, and elevated AST/ALT ratios are frequently observed despite low rates of clinically significant fibrosis [70, 71]. Regional variation in hepatotoxicity prevalence is notable, ranging from ∼12% in China to 37% in Cameroon, influenced by ART regimen type, coinfections, and genetic predisposition [69, 72, 73].

However, this finding should be interpreted cautiously because elevated AST/ALT ratios may arise from a variety of hepatic and extrahepatic factors, including age‐related physiological changes, muscle metabolism, chronic inflammation, medication exposure, and underlying liver disease. In the absence of imaging‐based assessments or histological confirmation, the clinical significance of these elevated ratios cannot be determined.

Noninvasive fibrosis indices supported the interpretation that most participants were at low risk of advanced liver fibrosis. The majority were classified as low risk by APRI and FIB‐4 scores, although a meaningful minority fell into intermediate or high‐risk categories. These findings are consistent with previous studies, demonstrating that APRI and FIB‐4 can detect early fibrosis and provide useful screening information in resource‐constrained settings [67, 74].

However, both indices have recognized limitations in PLWH. APRI may be influenced by HIV‐associated thrombocytopenia and other factors affecting platelet counts. Importantly, FIB‐4 incorporates age directly into its calculation; therefore, higher FIB‐4 scores observed among older participants may be partly driven by the mathematical structure of the index rather than reflecting a true increase in liver fibrosis burden. Consequently, age‐group differences in FIB‐4 should be interpreted with caution and should not be assumed to represent progressive fibrosis without confirmatory assessment. Furthermore, APRI and FIB‐4 are screening and risk‐stratification tools rather than definitive diagnostic measures of liver fibrosis. Although these indices are valuable in resource‐limited settings where imaging modalities may not be routinely available, elevated scores should not be interpreted as evidence of established fibrosis in the absence of confirmatory investigations such as transient elastography, ultrasound‐based fibrosis assessment, or liver histology. Accordingly, the fibrosis‐related findings reported in this study should be regarded as indicative of potential fibrosis risk rather than confirmed liver fibrosis. Several studies suggest that APRI may be more suitable for pediatric populations, although optimal cutoff values remain uncertain. Despite these limitations, longer duration and earlier initiation of ART have consistently been associated with lower APRI and FIB‐4 scores, supporting the protective role of effective viral suppression against liver fibrosis [71, 75, 76]. Total protein and globulin concentrations were higher in younger participants, while albumin remained stable across age groups, suggesting that protein abnormalities in PLWH primarily reflect chronic immune activation and polyclonal B‐cell stimulation rather than hepatic dysfunction [32]. The preservation of albumin indicates intact hepatic synthetic function. Serum albumin is a recognized prognostic marker in HIV infection, with hypoalbuminemia linked to advanced disease, low CD4 counts, high viral load, and increased mortality [77, 78]. Baseline hypoalbuminemia in African cohorts ranges from 19.6% to nearly 40%, especially among ART‐naïve or advanced‐stage patients [68]. The low prevalence in our study likely reflects early diagnosis, widespread ART use, and effective viral suppression, which improves albumin via immune reconstitution [71]. Given its low cost and wide availability, serum albumin remains a valuable surrogate marker for immune status and disease progression in resource‐limited settings, although its prognostic utility at the individual patient level remains debated [77].

In this study, the composite renal–hepatic–virologic abnormality score differed significantly across age groups among PLHIV, with adolescents aged 13–19 years and older adults aged ≥ 60 years exhibiting a greater burden of concurrent abnormalities than children aged ≤ 12 years. The increased burden among adolescents may reflect, at least in part, the complex transition period associated with adolescence, including challenges with long‐term ART adherence, psychosocial factors, and transition from pediatric to adult‐oriented HIV care. Previous studies have reported that adolescents living with HIV are particularly vulnerable to suboptimal treatment adherence and viral suppression challenges, which may contribute to persistent virologic abnormalities and downstream clinical complications [45, 79]. The higher abnormality burden observed among older adults is consistent with evidence that people aging with HIV experience increased risks of CKD, liver abnormalities, and other noncommunicable complications. Age‐related physiological changes, prolonged exposure to HIV infection, cumulative ART exposure, and increased prevalence of comorbidities may contribute to declining organ function and abnormal biochemical profiles in older PLHIV, although these factors were not directly assessed in the present study [8, 14].

The finding that most participants had at least one abnormality highlights the importance of evaluating HIV‐related health beyond viral suppression alone. A composite renal–hepatic–virologic abnormality score may provide a useful approach for identifying individuals with multiple clinical and biochemical concerns who may benefit from closer monitoring and targeted interventions.

The overlap observed among elevated AST/ALT ratio, reduced eGFR, and unsuppressed HIV viral load highlights the potential coexistence of hepatic, renal, and virological abnormalities within a subset of participants. However, the Venn diagram analysis was descriptive in nature and was not intended to identify causal pathways or independent predictors of these overlapping conditions. Similar clustering of renal, hepatic, and virological abnormalities has been reported in cohort studies, where poor viral suppression frequently co‐occurred with kidney and liver abnormalities, potentially reflecting shared pathways such as chronic immune activation and systemic inflammation [80–82]. This multisystem burden underscores the interrelated nature of HIV‐associated comorbidities, where poor viral suppression may coexist with renal and hepatic abnormalities through shared underlying mechanisms, including chronic immune activation and systemic inflammation. Future studies incorporating multivariable modeling and longitudinal follow‐up may provide a more comprehensive understanding of the factors associated with concurrent organ dysfunction and virological outcomes among PLHIV.

The findings of this study highlight several actionable strategies to strengthen clinical care and inform health policy for PLHIV in Ghana. Routine monitoring of renal and hepatic function should be systematically integrated into HIV treatment protocols, with particular emphasis on aging populations and individuals receiving long‐term ART. The incorporation of clinical decision‐support tools within ART clinics could facilitate early identification of declining eGFR or rising AST/ALT ratios, enabling timely clinical intervention. In addition, nutrition‐focused strategies should be reinforced, with longitudinal trends in serum albumin and total protein used to inform both individualized patient management and broader community‐level care planning. Strengthening collaboration across HIV, renal, and NCD services within the health system would enhance continuity and coordination of care. Finally, investment in longitudinal surveillance systems is essential to track biochemical trajectories over time, allowing early detection of subclinical organ dysfunction and prompt preventive or therapeutic responses.

### 4.1. Study Limitations

This study has several limitations that should be considered when interpreting the findings. First, the cross‐sectional design cannot establish causal relationships or determine temporal associations between aging, HIV‐related factors, ART exposure, and renal or hepatic outcomes. Second, the absence of an HIV‐negative comparison group limits our ability to distinguish age‐related physiological changes from effects potentially attributable to HIV infection or long‐term ART exposure. Third, information on several important clinical variables was unavailable or incomplete, including Hepatitis B and C coinfection status, alcohol consumption, duration of HIV infection, obesity, concomitant medication use, and detailed ART history. Although blood pressure measurements and selected comorbidity data were available, information on hypertension, diabetes, and other NCDs was limited, restricting our ability to fully adjust for potential confounding factors. In addition, ART duration data were only available for a subset of participants, limiting their inclusion in multivariable analyses. Fourth, assessment of renal function relied primarily on serum creatinine, urea, and eGFR, without urine‐based markers such as proteinuria, albuminuria, or urinary albumin‐to‐creatinine ratio, which may provide a more comprehensive evaluation of kidney injury. Similarly, liver fibrosis was assessed using APRI and FIB‐4 scores, which are noninvasive screening tools rather than definitive diagnostic measures. Interpretation of FIB‐4 warrants caution because age is incorporated directly into its calculation and may contribute to higher scores among older participants independent of underlying fibrosis severity. Furthermore, elevated APRI and FIB‐4 scores should not be interpreted as evidence of established fibrosis without confirmatory imaging or histological assessment. Finally, the possibility of survivor bias cannot be excluded, as older participants who remained engaged in HIV care may represent a healthier subset of the broader population of PLHIV. Despite these limitations, this study provides important data on age‐group differences in renal, hepatic, and virological profiles among PLHIV in Ghana. The ongoing longitudinal component of the GenoPharm‐GH cohort will enable future analyses incorporating additional clinical variables, genetic risk markers, and more comprehensive renal and hepatic assessments to better characterize trajectories of organ health across the lifespan.

## 5. Conclusion

This study identified important differences in clinical and biochemical profiles across age groups among PLHIV in Ghana. Younger participants generally exhibited better renal function and higher rates of virologic nonsuppression, while also demonstrating elevated globulin levels and modest alterations in liver‐related biomarkers.

Older participants had lower eGFR values, higher rates of viral suppression, and higher APRI and FIB‐4 scores, although interpretation of FIB‐4 should be made cautiously because age contributes directly to the score calculation. The findings highlight the importance of age‐sensitive and integrated HIV care that incorporates routine assessment of renal, hepatic, and nutritional health alongside virological monitoring. Strengthening biochemical monitoring within ART programs may facilitate earlier identification and management of organ‐related abnormalities and support the long‐term care of the growing population of PLHIV in Ghana and across SSA.

**Nomenclature**
ART: Antiretroviral therapy
HIV: Human immunodeficiency virus
PLHIV: People living with HIV
SSA: Sub‐Saharan Africa
AIDS: Acquired immunodeficiency syndrome
NADMs: Non–AIDS‐defining morbidities
NAFLD: Nonalcoholic fatty liver disease
ALT: Alanine aminotransferase
AST: Aspartate aminotransferase
ALP: Alkaline phosphatase
CKD: Chronic kidney disease
TDF: Tenofovir disoproxil fumarate
eGFR: Estimated glomerular filtration rate
SBP: Systolic blood pressure
DBP: Diastolic blood pressure
MAP: Mean arterial pressure
WHO: World Health Organization
FIB‐4: Fibrosis 4 index
APRI: Aspartate aminotransferase to platelet ratio index
SD: Standard deviation
ANOVA: Analysis of variance
CCTH ERC: Cape Coast Teaching Hospital Ethical Review Committee
GHS‐ERC: Ghana Health Service Ethical Review Committee
NCDs: Noncommunicable diseases
KDIGO: Kidney Disease: Improving Global Outcomes
A: Albumin
G: Globulin
OR: Odds ratio
aOR: Adjusted odds ratio
VL: Viral load
DTG: Dolutegravir

## Author Contributions

Oksana Ryabinina and Nicholas Ekow Thomford conceptualized this idea. Oksana Ryabinina, Nicholas Ekow Thomford, Joel Adu Twum, John Anyimadu, Hilary Kenneth Addison, Paul Jerrod Amoako, and Samuel Badu Nyarko were involved in recruitment. Joel Adu Twum, John Anyimadu, Hilary Kenneth Addison, and Paul Jerrod Amoako undertook laboratory analysis. Oksana Ryabinina undertook data analysis and wrote the first draft. Nicholas Ekow Thomford and Oksana Ryabinina worked on the draft. Oksana Ryabinina, Nicholas Ekow Thomford, and Samuel Badu Nyarko secured funding and support for the work. All authors worked to finalize the manuscript.

## Funding

Data for this study were obtained through funding from the EDCTP2 programme (TMA2019CDF‐2670) supported by the European Union and Novartis Global Health Basel Switzerland, the International Foundation for Sciences (I‐3‐F‐6441‐1), the National Institute for Allergy and Infectious Diseases (1R21AI186750‐01/02), and the National Research Foundation of South Africa through a rated research incentive award (UID127492) to Nicholas Ekow Thomford, Sam, and Brew Butler award from the School of Graduate Studies, University of Cape Coast, Ghana, to Samuel Badu Nyarko and reagent support funding by Oksana Ryabinina. The authors are responsible for the contents of this manuscript, and the funders have no role in the data and information provided.

## Disclosure

All authors approved the final manuscript.

## Ethics Statement

Ethical approval for the study was obtained from the CCTH ERC (CCTHERC/EC/2020/109; CCTHERC/EC/2021/005; CCTHERC/EC/2023/090) and the Ghana Health Service Ethical Review Committee (GHS‐ERC: 001/12/24). Permission to access clinic records was obtained from facility management. All data were anonymized, and patient identifiers were removed to ensure confidentiality in accordance with ethical research standards and the principles of the Declaration of Helsinki.

## Conflicts of Interest

The authors declare no conflicts of interest.

## Supporting Information

Additional supporting information can be found online in the Supporting Information section.

## Supporting information


**Supporting Information 1** Supporting Figure S1. Venn diagram showing overlap between uncontrolled HIV viral load (HIV VL not suppressed), elevated AST/ALT ratio (> 1), and reduced kidney function (eGFR < 60 mL/min/1.73 m^2^) among study participants (*N* = 473).

**Supporting Information 2** STROBE Statement—Checklist of items that should be included in reports of cross‐sectional studies.

## Data Availability

The datasets used and/or analyzed during the current study are available from the corresponding authors upon reasonable request.
