# Supplementary material for: Age‐Related Variations in Clinical and Biochemical Profiles of People Living With HIV
Source: AIDS Res Treat. 2026 Sep 26;2026:1670270. doi: 10.1155/arat/1670270 (PMC13615455; doi:10.1155/arat/1670270)
Supplement: Supplementary file 1 [file ARAT-2026-1670270-s001.docx]

**Supplementary file 1**

**Figure 1:** Venn diagram showing overlap between uncontrolled HIV viral load (HIV VL not suppressed), elevated AST/ALT ratio (>1), and reduced kidney function (eGFR <60 mL/min/1.73 m²) among study participants (N = 473). The diagram illustrates the distribution of single conditions and their co-occurrence across the three clinical parameters, with percentages indicating proportion of the total sample.
